# Ovaritis

**Published:** 1853-09

**Authors:** 


*Ovaritis.* By Dr. Coale.—The patient had suffered for seven years with symptoms referred by her attending physician to prolapsus uteri; there was violent pain at the menstrual periods, and also at other times; pain and sense of weight in the back, and pain of very acute character in the front of the abdomen darting back to the renal regions; an opiate treatment, alone, had been followed. On examination per vaginam, Dr. C. detected the right ovary enlarged, hardened, and very tender to the touch; there was but a *very slight* degree of prolapsus of the womb; an issue over the region of the inflamed ovary, and quinia were ordered. Dr. C. thinks this affection is very often overlooked. Relief was obtained by the above treatment.—*Am. Jour. Med. Sciences.*

